# Polyacrylamide-Metilcellulose Hydrogels Containing *Aloe barbadensis* Extract as Dressing for Treatment of Chronic Cutaneous Skin Lesions

**DOI:** 10.3390/polym12030690

**Published:** 2020-03-19

**Authors:** Desireé Alesa Gyles, Anivaldo Duarte Pereira Júnior, Lorena Diniz Castro, Andressa Santa Brigida, Maria Louze Nobre Lamarão, Wagner Luiz Ramos Barbosa, José Otávio Carréra Silva Júnior, Roseane Maria Ribeiro-Costa

**Affiliations:** 1 Laboratory of Pharmaceutical Nanotechnology, College of Pharmacy, Federal University of Pará, Belém 66075-110, Brazil; desiree.gyles@gmail.com (D.A.G.); anivaldoj@gmail.com (A.D.P.J.); louzelamarao@gmail.com (M.L.N.L.); 2 Laboratory of Pharmaceutical and Cosmetic R&D, College of Pharmacy, Federal University of Pará, Belém 66075-110, Brazil; lorenadiniz9@gmail.com (L.D.C.); carrera@ufpa.br (J.O.C.S.J.); 3 Laboratory of Chromatography and Mass Spectrometry, Faculty of Pharmacy, Federal University of Pará, Belém 66075-110, Brazil; andressabriggida@gmail.com (A.S.B.); barbosa@ufpa.br (W.L.R.B.)

**Keywords:** hydrogel, *Aloe barbadensis*, polyacrylamide, methylcellulose, skin lesions, phytotherapy

## Abstract

Chronic wounds are severe breaks in the skin barrier that fail to heal in an acceptable time-frame, thus preventing the complete restoration of the tissue’s anatomical and functional integrity, increasing the likelihood of infections and apoptosis. Hydrogels are known as a drug delivery system and have the potential to cover wounds and burns on the skin. *Aloe barbadensis* contains over 75 different bioactive compounds which are responsible for its anti-inflammatory and antimicrobial properties. In this study, the polyacrylamide-co-methylcellulose hydrogel containing *Aloe barbadensis* were developed. The extract was prepared from lyophilized *Aloe barbadensis*, using methanolic extraction, characterized by high performance liquid chromatography and incorporated into the hydrogels. These *Aloe barbadensis* hydrogels were characterized by degree of swelling, Fourier-transform infrared spectroscopy, scanning electron microscopy, and thermal profiling using thermogravimetric analysis. The minimum inhibitory concentration test was done on the *Aloe barbadensis* extract to evaluate its antibacterial and antifungal activity in vitro. The *Aloe barbadensis* hydrogels and were shown to swell to almost 2000% of their original sizes. The Fourier-transform infrared spectroscopy indicated the presence of bands characteristic of *Aloe barbadensis* and hydrogel polymers. The basic hydrogel showed greater thermal stability than the hydrogels with *Aloe barbadensis*. The minimum inhibitory concentration showed inhibition of the growth of *S. aureus* and *Salmonella* spp. at specific concentrations. The hydrogel therefore presents itself as an excellent potential curative cover of cutaneous lesions.

## 1. Introduction

Chronic wounds are severe breaks in the skin barrier that fail to heal in an acceptable time-frame, thus preventing the complete restoration of the tissue’s anatomical and functional integrity, increasing the likelihood of infections and apoptosis. Wounds become chronic if they do not pass through all the phases of healing, that include hemostasis, inflammatory, proliferative, and remodeling [1,2]. Chronic wounds are an unsuspecting epidemic that affects a large fraction of the world’s population [3]. Approximately 1–2% of the population of developed countries will suffer from the malady of a chronic wound during their lifetime, a probability that increases with age [4]. Various complications of these wounds, such as gangrene, hemorrhage, and lower-extremity amputations often result, causing further discomfort and debility of the patient [5]. As a result, chronic wounds pose a grave impact to the health and quality of life of patients and their families often resulting in pain, loss of function and mobility, emotional, social, and financial challenges, prolonged hospital stays, and chronic morbidity or even death [6]. In addition to the burden it places on the individual personally, the healthcare system and the society as a whole is also impacted [7,8]. In the United States alone, for example, chronic wounds are reported to affect 6.5 million patients, causing more than USD 25 billion to be charged to the healthcare system each year as a result of wound-related complications [3]. This expense is also reflected in the United Kingdom, where the resulting cost to the National Health Service in the care of these kinds of wounds were roughly estimated at USD 3.4–4.6 billion per year (in 2005), which was about 3% of the total estimated expenditure on health for the same year [9].

The healing of chronic cutaneous lesions requires an efficient transmission of information between the different cellular constituents of the skin and its extracellular matrix (ECM). This process of skin barrier repair is often efficient under normal physiological conditions but has the potential to become chronic in cases of impaired immunity and/or systemic factors. Chronic wounds are generally classified on the basis of causality, including: vascular insufficiency, diabetes mellitus, and pressure wounds. It is important to note that nutrition, compromised immunity, age, and mechanical stress among other factors can affect an individual’s ability to successfully heal these wounds [10]. However, the existing treatments are palliative and costly. There is a great need for more effective treatments, to make the healing process of these wounds more efficient. The most ideal curative treatments of cutaneous wounds should: improve healing time, reduce infections and contamination of microorganisms, relieve pain, allow gas exchange, and be of low cost [11]. Along this line, this hydrogel system was developed to have a drug delivery mode of action in order to promote the regeneration of skin, hair follicles, and glands while accelerating the healing process and reducing pain [12,13].

Hydrogels and their biomedical applications date back to 1950. In biotechnological advances they have been used in a variety of healing treatments and even in bone implants. The hydrogels have three-dimensional polymeric structures, that are classified as either homopolymerous or copolymerous [14]. The hydrophilic characteristic of the hydrogels is due to the presence of associated hydrophilic molecules, which enable them to be able to absorb up to 90% of their weight in fluids. When swollen hydrophilic network systems also have the potential to mimic biological tissues, a characteristic which helps in their biomedical application [15].

Among the most used polymers for the synthesis of hydrogel, polyacrylamide (synthetic), and methylcellulose (natural) are highlighted. Polyacrylamide has low toxicity and a low sourcing cost. Its synthesis occurs by free radical polymerization in aqueous solution or polymerization of crystalline acrylamide in the solid state with ionizing radiation. The acrylamide polymers and their derivatives are well known for their hydrophilic and inert nature, which makes them suitable for biomedical applications [16,17]. On the other hand, methylcellulose is a natural polymer that is derived from cellulose by replacing the hydroxyl groups with methyl groups [16,18]. In this way, the mixture of the synthetic and the natural polymers in the synthesis of hydrogels aims to improve the mechanical structure and to provide the biodegradability.

Associated with the hydrogel technology was the incorporation of *Aloe barbadensis*, an herb traditionally used in wound treatment to further enhance the healing properties of the hydrogel [19]. *Aloe barbadensis* is a plant also commonly referred to as Aloe belonging to the family Liliaceae, which has been used for centuries in products aimed at health, beauty, and care of the skin [20,21,22]. This herb is a succulent plant that originates in India, Africa, the Caribbean, Mediterranean, and other arid regions of the world. It has a high concentration of water ranging from 99% to 99.9%, and 1% solid material containing more than 75 different bioactive substances that possess: anti-inflammatory properties, laxative effects, antiviral, antibacterial, anti-fungal activities, and anti-aging properties.

*Aloe barbadensis* stands out as a king among herbs for accelerating the healing of wounds and the reduction of scarring [23,24]. Many of its medicinal effects have been attributed to polysaccharides found in the inner leaf parenchyma. However, many researches also indicate higher antioxidant activities inside its outer skin due to the presence of anthraquinones [23]. The most researched medicinal components of *Aloe barbadensis* are barbaloin, glucomannan, and aloe-emodin. Glucomanan is a polysaccharide rich in mannose—a growth hormone that interacts with fibroblast growth factor receptors in wounds—thus stimulating their activity and proliferation [25]. This action significantly increases the synthesis of collagen, along with the action of mannose, which is known to promote wound healing and to rebuild collagen tissue [26]. Barbaloin, also called aloin, is a C-Glucoside found in the outer layer of the plant. It has cathartic, antioxidant, and anti-inflammatory properties in in vivo tests [26].

Using the concept mentioned above, this study proposes the association of the innovative hydrogel technology with the technological development of phytotherapy by obtaining a polyacrylamide–methylcellulose hydrogel containing *Aloe barbadensis*, to develop a bandage for the treatment of chronic cutaneous lesions.

## 2. Materials and Methods 

### 2.1. Materials and Reagents 

The leaves of *Aloe barbadensis* (Exsiccata no. IAN 194.547) were collected from the Horticulture of the Brazilian Agricultural Research Corporation (EMBRAPA) of the Eastern Amazon, in the State of Pará, Brazil. Acrylamide was purchased from Vetec^®^ (Rio de Janeiro, Brazil); methylcellulose (viscosity 2%), *N,N*-methylene-*bis*-acrylamide, *N,N,N,N*-tetramethylethylenediamine (TEMED) and Aloin were obtained from Sigma Aldrich^®^ (Darmstadt, Germany). Ultra-pure water was obtained using a Millipore water system MilliQ^®^ (Sigma Aldrich^®^, Darmstadt, Germany); Acetonitrile was obtained from Tedia^®^ (Rio de Janeiro, Brazil). Microbial cultures from obtained from the American Type Culture Collection (*Staphylococcus aureus* (ATCC 29213), *Escherichia coli* (ATCC 25318), *Salmonella enterica* (ATCC 1408), *Enterobacter aerogenes* (ATCC 13048), and *Candida albicana* (ATCC 14053)) were obtained from the Oswaldo Cruz Foundation (FIOCRUZ) and provided by the State Central Laboratory (LACEN-PA).

### 2.2. Preparation of Aloe barbadensis Plant Material

The leaves of *Aloe barbadensis* were cut longitudinally with a knife, and the clear gel was removed with a spatula [27]. The gel was then homogenized in a blender and separated into two aliquots. One part was transferred to a glass receptacle for freezing at −18 °C, which would be later lyophilized and used for preparation of the extract, and the other part was used for the physicochemical characterization of the gel *in natura*.

### 2.3. Preparation of Aloe barbadensis Extract

The preparation of the extract from lyophilized *Aloe barbadensis* (AL) was performed by the method adapted from Azaroual et al. (2012) [28]. Two (2) g of AL was weighed and placed into a glass beaker, where 50 mL of ultrapure water and 50 mL of methanol (50% *v/v*) were added to form a 2% solution. The solution was placed into an ultrasonic bath for 15 min at 37 °C, and subsequently left for 24 h at room temperature. After this step, the methanol was removed using the Buchi^®^ rotary evaporator (Flawil, Switzerland) at 30° C and the aqueous phase was subjected to centrifugation at 11,000 rpm for 15 min in an Eppendorf^®^ centrifuge (Hamburg, Germany). The supernatant, from then on was called the *Aloe barbadensis* extract (EA) and was collected for characterization by HPLC-DAD-EM, and preparation of the hydrogels.

### 2.4. Physical–Chemical Characterization Assays of Aloe barbadensis 

The following physico–chemical characterization tests were performed with *Aloe barbadensis in natura* and EA.

#### 2.4.1. pH Analysis of *Aloe barbadensis*

The pH determination was performed with a pH potentiometer HANNA^®^ (Sao Paulo, Brazil), that was previously calibrated with standard buffer pH 4.0 and 7.0 and the results corresponded to the average of three independent determinations.

#### 2.4.2. Relative Density Analysis

The relative density was done using a 5 mL pycnometer, which was filled with the standard liquid (distilled water free of carbonic gas) and weighed. The pycnometer was then filled with 5 mL of the sample and weighed. The assay was performed in triplicate [29].

#### 2.4.3. Fiber Content and Moisture Analysis

To determine the percentage of water and insoluble solids,10 mL triplicate samples of the *Aloe barbadensis* extract (EA) sample were weighed. The samples were then placed in glass receptacles and the weight verified again, after which they were placed in the laboratory oven at 45 °C for 24 h or until the samples were completely desiccated. After this period, the receptacles were removed from the oven, cooled and weighed. The result was calculated using Equation (1).

(1) $$\%TA=\frac{A-B}{B}\times 100\%$$

where: *%TA* represents the percentage of total water, *A* represents the wet weight of the sample, while *B* represents the dry weight of the sample and the weight of the insoluble fiber [27].

#### 2.4.4. Analysis of the Extract of *Aloe barbadensis* by Liquid Chromatography of Ultra Efficiency Coupled to Mass Spectrometry (HPLC-DAD-EM)

The identification of Aloin from EA was done by comparing the samples tested with the aloin standard (Sigma^®^ (natural mixture of diastereomers aloin A (barbaloin) and B (isobarbaloine)), compared with the UV/MS spectra, as well as by co-elution with authentic standard. The analyzes were performed using an Agilent system consisting of a UHPLC model 1290 with column oven (G1316A), diode arrangement detector (DAD) (G1315C), automatic sampler (G4226A) and quaternary pump (G4204A) coupled to a triple quadrupole mass spectrometer (QQQ) model 6460C (Agilent Technologies, Sao Paulo, Brazil). A Zorbax Eclipse XDB-C8 reverse phase column with flow rate of 0.4 mL/min, injection volume 5 μL and maximum pressure 400 bar was used. The mobile phase was composed of water acidified with formic acid (pH 3) (Solvent A) and acetonitrile (Solvent B). Elution of the solvents took place in the following gradient: 0–7 min 10% B, 7–8 min 10–22% B, 8–14 min 22% B, 14–18 min 22–50% B, and 18–33 min 50–95% B. The detection wavelength ranged from 210 nm–400 nm as detection at 254, 280, 315, 330, and 350 nm. The column temperature was 35 °C. In the mass spectrometer the analyzes were done with N_2_ flux of 5 L/min, electrospray ionization source temperature (ESI) of 300 °C, 45 psi nebulizer, 3500 V capillarity, and 80 V shredder voltage.

### 2.5. Preparation of Hydrogel Containing Extract of Aloe barbadensis

The polyacrylamide-methylcellulose hydrogels containing the *Aloe barbadensis* extract (HD-PM-EA) were obtained by free radical polymerization [30]. For the synthesis of the hydrogel, 7.2% (*w/v*) of acrylamide (AAm) and 0.5% (*w/v*) of methylcellulose (MC) were used. The procedure was initiated by the addition of the monomers AAm and MC, following the addition of the crosslinking agent *N’-N*-Methylenebisacrylamide (MBAAm) with a concentration of 8.55 μmol·mL^−1^. Then the *N,N,N‘,N’*-Tetramethylethylenediamine (TEMED) catalyst at a concentration of 3.21 μmol·mL^−1^ and the sodium persulfate (PS) concentration of 3.38 μmol mL^−1^ were added to the reaction, the latter being the initiator of the polymerization reaction. Thirty milliliters of the EA was added to the polymer mixture to synthesize the 2% hydrogel (2% HD-PM-EA). Fifteen (15) mL extract and 15 mL ultrapure water (50%, *v/v*) were added the mixture to synthesize the 1% hydrogel (1% HD-PM-EA). The total volume of the hydrogels was 30 mL. The percentage of hydrogels was allocated based on the percentage of bioactive solids EA, relative to the hydrogel.

All components were homogenized in a beaker and the reaction carried out in N_2_ atmosphere for 20 min to eliminate the oxygen until complete polymerization of the reaction occurred, forming the hydrogel. The polyacrylamide-methylcellulose hydrogel without the *Aloe barbadensis* extract was called the basic hydrogel (HD BA) [31].

The HD-PM-EA were washed in distilled water (100 mL) for three days (with water change) in order to remove excess chemical elements that did not form within the hydrogel matrix. Subsequently, the HD-PM-EA were oven dried at 45 °C, then micronized in a Fritsch 14 Micronizer (Fritsch^®^, Idar-Oberstein, Grmany) with a mesh of 500 μm for characterization tests.

### 2.6. Physical–Chemical Characteristics of Hydrogel containing Aloe barbadensis Extract

#### 2.6.1. Determination of Degree of Swelling

HD-PM-EA at concentrations 1% and 2% and HD BA were weighed to obtain initial weight and then immersed in 100 mL distilled water at 37 ± 0.5 °C. HD-PM-EA was removed from the medium at specific time intervals (0.5, 1, 3, 6, 24, 48, 72, 192, 336, 360, and 528 h), excess water removed from its surface, and weighed. The tests were done in triplicates and the degree of swelling was obtained by the ratio between the mass of the swollen hydrogels and the mass of the dry hydrogel [14], shown in Equation (2).

(2) $$Q\%=\frac{M_{t}}{W_{0}}\times 100$$

where *M_t_* is the mass of the swollen hydrogel and *W*_0_ is the mass of the dry hydrogel, after drying.

#### 2.6.2. Morphology of Hydrogels through the Scanning Electron Microscopy (SEM)

The dried, micronized samples were deposited on carbon double-sided adhesive tape, affixed to the carrier and then metallized with gold. The micrographs of the hydrogels were obtained using the Scanning Electron Microscope VEGA3 (Tescan^®^, Fuveau France) [32,33].

#### 2.6.3. Analysis of Formulation Excipient Profiles

The Fourier Transform Infrared Spectroscopy (FTIR) (Agilent Technologies, Sao Paulo, Brazil) was used to evaluate the presence of the functional groups of the three-dimensional structure of the obtained hydrogels. In this assay, the samples used were: AL, HD BA, and 1% and 2% HD-PM-EA. The dried, micronized samples were mixed with potassium bromide (KBr) and pressed under high pressure forming tablets, which were analyzed [34,35]. The absorption spectra were evaluated in the range of 4000 to 400 cm^−1^, with 20 scans and 2 cm^−1^ resolution [32].

#### 2.6.4. Thermogravimetric Profile

Thermogravimetric analysis was performed on samples AL, HD BA, and HD-PM-EA 1% and 2%. Five (5) mg of each sample was weighed in a platinum crucible and subjected to a temperature of 25–600 °C with a heating rate of 10 °C/min under a nitrogen atmosphere (N_2_) in a thermobalance Shimadzu TGA 50 (Kyoto, Japan) [36].

### 2.7. In Vitro Activity Test of Antibacterials and Antifungals

#### 2.7.1. Preparation of Media and Biological Material

The media (Sabouraud Dextrose Agar, Cetrimide Agar, MacConkey Agar, Mannitol Agar, Müller Hinton Agar and Müller Hinton Broth) were prepared according to manufacturers. Initially, the medium of the sterile agar was distributed in Petri dishes 15 × 90 (20 mL/plate), obtaining a layer of 4 mm thickness. After solidification the medium was exposed to the ultraviolet (UV) lamp for 15 min and then stored in a refrigerator at 4 °C. For the antimicrobial activity tests, standard strains of *Staphylococcus aureus, Escherichia coli, Salmonella enterica, Enterobacter aerogenes,* and *Candida albicans*, from the American Type Culture Collection (ATCC) were used.

#### 2.7.2. Determination of Minimum Inhibitory Concentration (MIC) of *Aloe barbadensis* Extract

The EA was submitted to microdilution for determination of MIC and minimum bacterial concentration (MBC) using the concentrations (1000, 500, 250, 125, 62.5, and 31.25 μg/mL). In 180-well plates, 180 μL of Müller Hinton broth was added, a 10 μL sample aliquot was added to the dilutions and 10 μL of the bacterial suspensions, totaling 200 μL.

As a positive control for the assays, gentamicin was used. As a negative control, 10 μL of the bacterial suspension +190 μL of Müller Hinton broth were placed in 3 wells and in another 3 wells, 10 μL of the bacterial suspension +10 μL of 70% alcohol and 180 μL of Müller Hinton broth, each done in triplicate. For each active sample the strains were prepared, two plates, one for the MIC test and the other for the MBC test [37].

After sowing, the plates were covered and incubated at 35 ± 2 °C for 24 h. After that time, 10 μL of a 3-[4–dimethylthiazole-2-yl]-2,5-diphenyltetrazolium bromide (MTT) solution (2 mg/mL) was added, the plate being again incubated for 3 to 4 h. MTT is a tetrazolium salt, which has yellow initial staining, however, in viable cells this salt is reduced to formazan crystals, which shows a blue stain [38,39].

For the classification of the bacteriostatic antimicrobial activity of EA and its fractions, the following minimum inhibitory concentration (MIC) parameters were adopted: high antimicrobial activity would be considered <100 μg/mL; MICs between 100 and 500 μg/mL were considered moderate; CIM values between 500 and 1000 μg/mL were considered weak and the sample considered inactive if the MIC > 1000 μg/mL [40,41]. After the MIC results, the samples were selected for the CBM test, where reversibility was assessed.

## 3. Results and Discussions 

### 3.1. *Aloe barbadensis* Extract

The EA was obtained from the *Aloe barbadensis* gel *in natura* due to the fact that it contains some of the bioactive compounds of the plant. The gel *in natura* has a high water content of 99%. The remaining 1% solid material, possesses more than 75 different bioactive substances, with antioxidant properties, laxative effects, anti-inflammatory, anti-viral, anti-bacterial, and anti-fungal properties [42,43,44,45].

EA was obtained through an extraction process involving methanol and ultrapure water (50%, *v/v*). Various solvents were tested for aloin extraction during the course of this investigation, and the aloin content was found to be higher with methanol extraction than with the other solvents. A probable cause is due to the fact that *Aloe barbadensis* is rich in anthraquinones which are polar and require highly polar solvents (such as methanol) for their extractions [46]. The higher the polarity of the solvent, the higher the extraction potential.

After the removal of the methanol using the rotary evaporator, the resultant extract was remained in the aqueous phase, which according to the literature contains a high level of aloin [47]. The extract had a dark yellow coloration with a slightly viscous consistency. From this extraction, the HD-PM-EA were synthesized.

This section may be divided by subheadings. It should provide a concise and precise description of the experimental results, their interpretation as well as the experimental conclusions that can be drawn.

### 3.2. Physical–Chemical Characterization Cssays of Aloe barbadensis 

The *Aloe barbadensis* gel *in natura*, is a transparent mucus-like, gelatinous mucilage, obtained from the leaves of the plant. This gel was characterized *in natura* in relation to pH, density and humidity. The physico–chemical characterization results of *Aloe barbadensis in natura*, and EA are summarized in Table 1.

The *Aloe barbadensis* gel *in natura* had a pH of 4.19, a result which falls within the parameters found in the literature (approximately 3.5–4.7) [44]. EA had a pH of 4.65 which is slightly higher than *Aloe barbadensis in natura* but is still within the parameters mentioned above. The probable cause for this pH can be attributed to the premature harvest time. In the moisture assay, the *Aloe barbadensis* gel *in natura* was found to be highly hydrophilic with 99% water and its relative density was 1.012 g/mL, greater than the density of pure water, which may be as a result of the insoluble fiber content of the gel which was 0.6%. On the other hand, EA had a density of 1.002, which is lower than *Aloe barbadensis* gel *in natura*, the probable being also related to the fiber content, which were removed during the extraction process.

According to the literature, the active components of *Aloe barbadensis* include: anthraquinones, vitamins, antioxidants, and glycoproteins, among others that are present precisely in the liquid portion of the gel and, thus, in its entirety. These components contribute to the pharmacological properties for which *Aloe barbadensis* is known, however, their concentrations may differ according to climate, environmental stresses and other conditions of growth [44].

### 3.3. Analysis of the Extract of Aloe Barbadensis by Ultra High Performance Liquid Chromatography Coupled to Mass Spectrometry (HPLC-DAD-EM)

**Figure 1:**
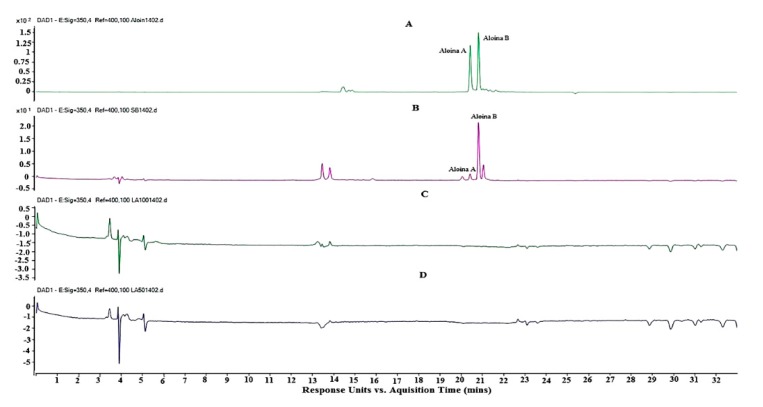
Chromatograms of Aloin (**A**), *Aloe barbadensis* extract (EA) (**B**), and washing of polyacrylamide-methylcellulose hydrogels containing the *Aloe barbadensis* extract (HD-PM-EA) 2% (**C**), and 1% (**D**).

**Figure 2:**
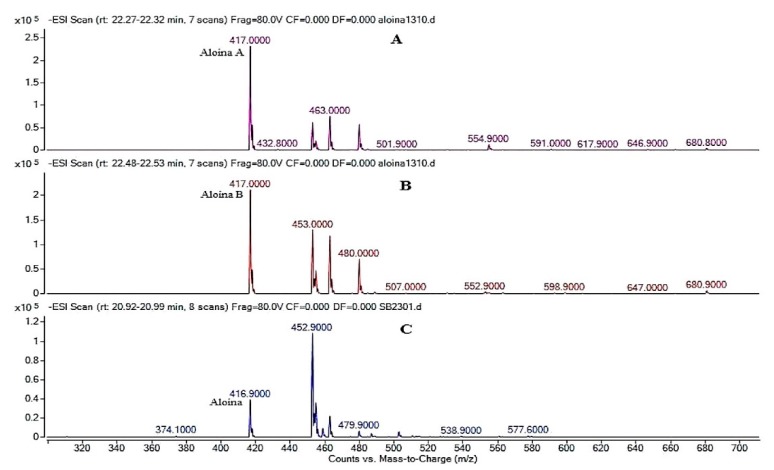
Mass spectrum of Aloin A (**A**), Aloin B (**B**), and EA (**C**).

**Figure 3:**
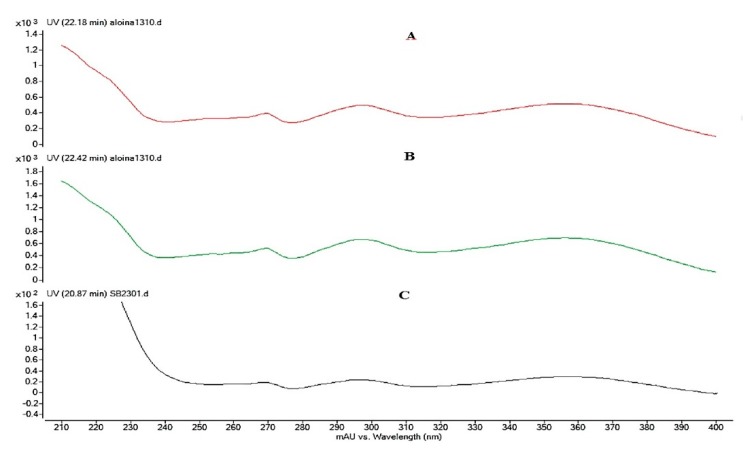
UV spectrum of Aloin A (**A**), Aloin B (**B**), and EA (**C**) with a wavelength of 350 nm.

The presence of Aloin in the EA was confirmed by comparing the retention times (*T*_r_) of the sample with the *T*_r_ of the Aloin standard (Sigma) (Figure 1), with the comparison of both the Ultra-violet (UV) spectra (Figure 2) and masses (Figure 3). This assay was conducted to quantify the amount of Aloin present in the EA. This is necessary to note because Aloin is one of the most important bioactive compounds in the *Aloe barbadensis* plant that gives accounts for its phytotherapeutical properties. 

Figure 1A shows the chromatogram of the Aloin A and B (barbaloin and isobarbaloin) standard, which shows two peaks: the first equivalent to Aloin A with a *T*_r_ of 20.5 min and the second peak of Aloin B with 20.8 min. *T*_r_ of Aloin A and B (Figure 1B) present in the EA were 20.5 min and 20.8 min, respectively. In both chromatograms, standard and sample (1A and 1B), higher levels are presented for Aloin B, especially in the EA extract sample. This result corroborates those of the investigations of Logaranjan et al. [48,49], in the quantification of aloe by HPLC from the *Aloe barbadensis* extract.

After the synthesis, HD-PM-EA 1% and 2% were subjected to the washing process to remove the excess of the chemical groups that did not participate in the formation of the polymer matrix. Water from this process was collected and analyzed by HPLC-DAD-EM, to evaluate if loss of EA and more specifically, Aloin occurred. Thus, Figure 1c,d show the chromatograms representing the wash water analyzes of 1% and 2% HD-PM-EA, respectively, which shows the absence of the albumin marker. These results suggest that there was interaction between the three-dimensional hydrogel matrix with *Aloe barbadensis*.

The molar mass of Aloin is 418.39 g/mol, which is more than the mass determined in the chromatograms in Figure 2. The formula of the aliphatic compound is 417 m/z, caused by the loss of an H^+^ that could be visualized both in the mass spectrum of the standard and in the extract of *Aloe barbadensis*. The mass spectrum chromatogram of the extract showed a unique peak of Aloin, due to the fact that Aloin A and B are diastereomers that have the same molar mass but have different chemical configurations [48]. The results (Figure 3), show that the peaks have a UV spectra wave length of 350 nm, which correspond to that of the standard Aloin, thus indicating its presence.

### 3.4. The Aloe barbadensis Hydrogel Synthesis

HD BA and HD-PM-EA 1% and 2%, were synthesized using the free radical polymerization. All hydrogels had a gelatinous, translucent appearance, while the hydrogel having 2% EA concentration showed a deeper yellow color than the hydrogel with 1% EA.

### 3.5. Physical–Chemical Characterization of Hydrogels

#### 3.5.1. The Swelling Analysis

The degree of swelling is a parameter used to characterize the three-dimensional hydrogel network. The ability of water absorption in the hydrogels is inversely proportional to the concentration of the polymer, which indicates that the lower the concentration of the polymer the greater the swelling degree [50,51].

**Figure 4:**
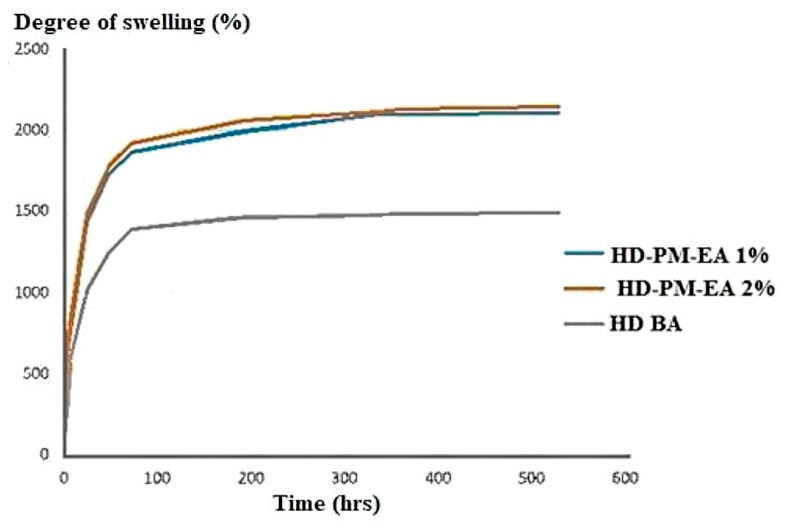
Degree of swelling of HD-PM-EA 2%, HD-PM-EA 1%, and basic hydrogel (HD BA).

The polyacrylamide polymer absorbs water by the formation of hydrogen bonds, through the mechanism of osmosis [50], however, the higher the concentration of the polymer the stronger the bonds formed within the network and the less its degree of swelling. It was observed that the HD-PM-EA profile had a degree of swelling of 2000%, that was approximately 1.34-times greater than the HD BA (1491%) (Figure 4).

HD-PM-EA had a higher degree of swelling due to the hydrophilic nature of *Aloe barbadensis*. The mucilaginous gel of the plant contains 99% water, and consequently, when associated with hydrogels that also have a large capacity to absorb water, the resulting hydrogels have a higher degree of swelling [43,44].

#### 3.5.2. Scanning Electron Microscopy (SEM)

The morphological characterization of AL, HD BA, and HD-PM-EA was determined through Scanning Electron Microscopy (SEM). SEM can be used to provide information on sample surface topography, composition, and other properties, such as electrical conductivity, so this technique is widely used to capture the characteristic structure of the three-dimensional ‘network’ of hydrogels which helps to determine its encapsulation potential [51,52].

**Figure 5:**
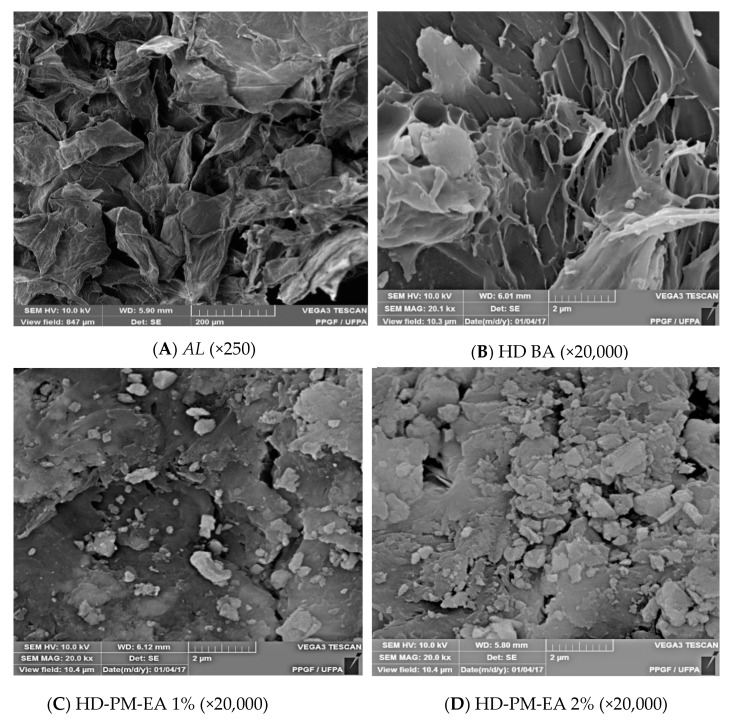
Photomicrographs of *Aloe barbadensis* (AL) (**A**), basic hydrogel (HD BA) (**B**), HD-PM-EA 1% (**C**), and 2% (**D**).

Figure 5 shows the photomicrographs of AL, HD BA, and 1% and 2% HD-PM-EA. In the photomicrograph of AL (Figure 5A) it was possible to verify the irregular fibrous cell wall structure. The morphology of HD BA (Figure 5B) shows a foliate structure typical of the three-dimensional network formed by the crosslinking agent. In the photomicrographs of HD-PM-EA 1% (Figure 5C) and 2% (Figure 5D), the fills of the wells formed by the pores of the polymer chain with the extract of *Aloe barbadensis* are shown, which represents the incorporation of the extract into the hydrogel matrix.

#### 3.5.3. Spectroscopic Profile By FTIR

**Figure 6:**
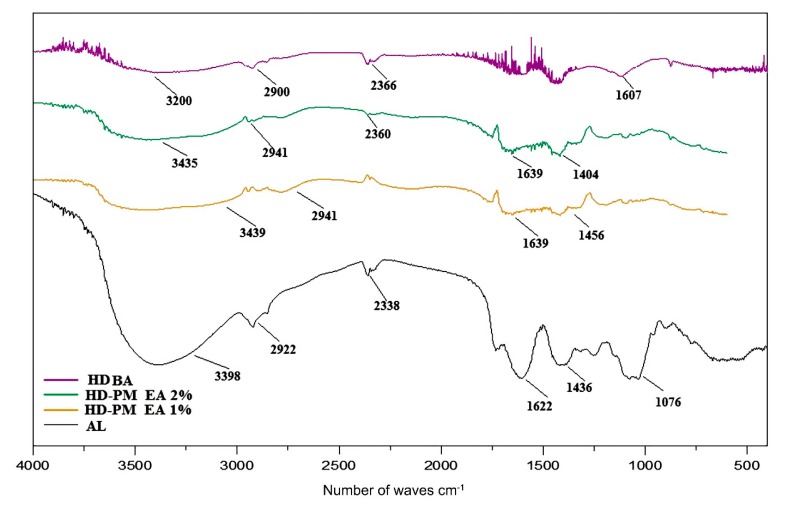
FTIR spectra of AL, 1% HD-PM-EA, 2% HD-PM-EA, and HD BA.

The spectroscopic profile in the infrared region with Fourier transform, besides providing characteristics of the compounds of the HD-PM-EA, aid in the visualization of the union of the copolymers. Figure 6 shows the FTIR profiles of AL, HD BA, and 1% and 2% HD-PM-EA. The use of AL and HD BA were used as control samples to identify the standard components of the *Aloe barbadensis* sample and those of the compounds within the hydrogel itself.

The profile of HD BA ratifies the presence of the functional groups characteristic of the monomer, with axial stretching, 3600–3200 cm^−1^ (axial deformation), 1607 cm^−1^ (angular deformation), indicating the presence of the group –OH, –NH–, and hydrogen carbon. Strong bands around 2900 cm^−1^ appear in almost all spectra of organic compounds, as they are due to the presence of the C–H stretch.

Table 2 indicates all the functional groups which are prominently featured in the HD-PM-EA. However, HD BA did not contain the groups: aromatic alkenes, bends and stretches, aldehydes, and ketones. It can therefore be deduced that these groups are unique to the composition of *Aloe barbadensis*, a fact which is confirmed when viewing the *Aloe barbadensis* profile. Therefore, 1% and 2% HD-PM-EA had the following functional groups: amines, alcohols, alkanes, and phenols. These functional groups indicate the presence of the medicinal components of *Aloe barbadensis*, such as: anthraquinones (barbaloin and emodin), saccharides, and vitamins B1, B2, B6, C, and A [42].

#### 3.5.4. Thermogravimetric Profile

Thermogravimetry (TG) is a widely used tool in the characterization of polymeric materials. Its micro-thermobalance measures any change in the mass of the sample associated with, oxygen adsorption, thermal degradation, oxidation, or other reactions. This change in the mass of the sample may indicate degradation, and in some cases it can also refer to increase of mass [50,53].

This technique of TG, is used in pharmaceutical science during pre-formulation or as a quality control to determine the thermal profile of drugs, which helps to determine their application, storage, and other properties [50]. 

**Figure 7:**
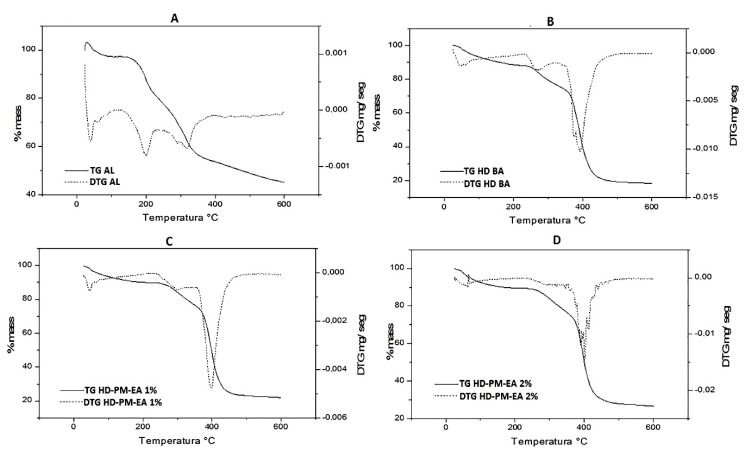
The thermograms of: (**A**) AL; (**B**) HD BA; (**C**) HD-PM-EA 1%; and (**D**) HD-PM-EA 2%.

The thermogravimetric (TG) analysis curves of AL, HD BA, and HD-PM-EA are shown in Figure 7. The TG curve of *Aloe barbadensis* (Figure 7A) showed four events, the first event occurred with a temperature range between 50–150 °C, with a mass loss of 46% resulting from the evaporation of water and volatiles. The second event occurred between 150–220 °C with a 14% loss of mass, which may be related to a decomposition of organic compounds, such as vitamins, minerals, antioxidants, polysaccharides, and amino acids content [54]. The third event occurred between 220–440 °C with a mass loss of 24% where fiber degradation began. The last event occurred between temperatures of 350–600 °C with a mass loss of 10% where the pyrolysis of the fibers occurs. 

The TG curve of HD BA (Figure 7B), which contains a mixture of acrylamide (AAm) and methylcellulose (MC) polymers demonstrated the characteristics of the components. The first event occurred between 30–250 °C with a mass loss of 10%, responsible for the loss of water mass and volatiles present in the sample. The second event was a successive degradation of the first, occurring in the range of 250–370 °C where 14% of the mass was lost. In the third event a mass loss of 48% was observed in the range of 350–430 °C. 

The reaction concluded with the final event, which began at 430 °C and ended at 600 °C without mass loss. The total mass loss of the basic hydrogel accompanied by the TG curve was 72%.

The thermograms of both the HD-PM-EA in the first event showed similar thermal behavior for both hydrogels with 10% mass loss and the same onset temperature. However, endset temperatures varied for both HD-PM-EA 1%, which began the second phase of degradation at 300 °C hydrogel. HD-PM-EA 2% showed a rapid loss of mass at 250 °C than 1% HD-PM-EA (Figure 7C), which demonstrated a thermal stability higher than 2% HD-PM-EA (Figure 7D).

Around 400 °C, degradation of the hydrogel resulted in residue (ashes). In the TG HD-PM-EA curves 1% and 2% show four main events, resulting in a mass loss of 75% and 80%, respectively. It has been observed that the thermal stability of AL was higher when incorporated into the polymer matrix of the hydrogel.

HD BA showed higher thermal stability when compared to HD-PM-EA and AL. According to Gabbay Alves et al. (2011) [50], polymers such as AAm and MC undergo degradation changes such as cracking and distortion as a result of external stimuli such as heat to break the polymer matrix. Therefore, the higher the polymer crosslinking, the greater the thermal stability. In addition, the loss of mass obtained resulted from loss of moisture and solvents used in its synthesis.

The TGA curves correspond to a degradation of the sample through a pyrolysis reaction, resulting in a residue (ash). In the case of HD-PM-EA, the percentage of ash was higher than that of HD BA, since AL has a higher concentration of minerals and other inorganic compounds [55,56]. Consequently, HD-PM-EA 2% would also have a higher ash percentage than HD-PM-EA 1%. Correspondingly, HD BA showed the smallest amount of ash residue when compared to the other samples.

According to Miranda et al. (2010) [57], *Aloe barbadensis* is known to lose its medicinal properties at temperatures of 80–90 °C. Mbese and Ajibade [58] and Dhakshnamoorthy et al. [59] indicate that the evaluation of the thermal stability in the TGA curves is related to a lower mass loss at high temperatures. Due to the fact that the proposed use of the formulation HD-PM-EA, is as a cover for cutaneous lesions at body temperature (36 °C), the formulation is predicted to not degrade, since it is thermally stable at this temperature.

#### 3.5.5. Evaluation of In Vitro Antibacterial and Antifungal Properties: Minimal Inhibitory Concentration (MIC) of *Aloe barbadensis*

Minimum inhibitory concentration in combination with the microdilution method is an in vitro technique that is able to demonstrate the lowest sample concentration required to inhibit microbial growth [60]. The results obtained are shown in Table 3.

Table 3 shows that at the concentrations used, three of the five strains showed no inhibitory activity. However, Salmonella had a minimum inhibitory activity at a concentration of 62.25 μg/mL and *S. aureus* also at the minimum inhibitory concentration at 250 μg/mL. When the samples were withdrawn from the wells the two strains (*S. aureus and Salmonella* spp.) Tested using the MIC method, no bacterial formation on the plaques was observed, confirming the inhibition of growth.

*Salmonella enterica* spp. is a Gram negative bacterium responsible for diseases such as food poisoning and typhoid, among others. However, in rare cases it is also known to cause rashes and ulcers particularly in immunocompromised patients [61,62]. Likewise, *Staphylococcus aureus (S. Aureus)* is a Gram positive bacterium that has the ability to cause food poisoning or skin infections based on the mode of transmission. Some symptoms of *S. aureus* skin infections include: benign boils, folliculitis, and more severe and invasive soft tissue infections [63]. Therefore, the inhibitory capacity of the *Aloe barbadensis* extract for these two pathogens is advantageous for its biomedical application in many areas, including the focus of this study—wound healing. One of the main complications of chronic wounds is microbial infection that can delay wound healing and worse yet, can cause sepsis and death [64].

Antibiotic and multi-drug resistance is a global concern for medical facilities, such as hospitals, sanatoriums, and other centers of healing and rehabilitation. These antibiotic resistance issues promote infections that often retard wound healing [65]. *Aloe barbadensis* is one of the most researched medicinal plants and is known to researchers for its antibacterial and antifungal activities in a wide range of gram positive and negative microbial species. According to Banu et al. [66], the in vitro and pasteurized *Aloe barbadensis* gel, when tested in resistant bacteria (including *Staphylococcus aureus and Enterobacter*) on multiple drugs in in vivo studies of infected leg ulcers, demonstrated a success rate of 93.3%. When evaluated in in vitro assays, Kumar et al. (2015) [61] also revealed that methanol-extracted *Aloe barbadensis* was effective in inhibiting the growth of organisms such as *E. coli* and *Salmonella*.

Several previous researches demonstrate that the concentration of minerals and phytochemicals present in medicinal plants depends to a large extent on factors such as geography, climate, seasonal changes, and soil fertility, among others. According to the study by Kumar et al. (2011) [67] the highest antimicrobial activity resulted from *Aloe barbadensis* plants found in regions of high environmental stress, such as higher altitudes and lower precipitation. *Aloe barbadensis* plants can grow practically anywhere, in any climate and yet have very effective medicinal properties. However, in regions with high environmental stress, more phytochemicals are produced in plants to combat this stress, which explains the higher medicinal properties of *Aloe barbadensis* plants grown in these regions [66,67]. The city of Belém do Pará/Brazil is located in a tropical forest region. As a result, it has an average temperature of 26.8 °C and an average rainfall of 2600 mm per year. Therefore, it is suggestive that the *Aloe barbadensis* plants harvested have a lower concentration of anthraquinones and other phytochemicals, due to environmental contributions, that would be necessary to reveal higher inhibitory activities [68].

## 4. Conclusions

The physico–chemical quality control of the *Aloe barbadensis* gel *in natura*, obtained from the leaf of *Aloe barbadensis*, presented results within the parameters found in other research literature. The extract of *Aloe barbadensis* (EA) made from the lyophilized gel (AL) showed the presence of the markers of the species, Aloin A and B, by HPLC-DAD-MS. From this finding, two polyacrylamide-co-methylcellulose hydrogels containing the *Aloe barbadensis* extract (HD-PM-EA) were prepared in concentrations of 1 and 2%.

The characterization of the hydrogels evidenced the presence of the *Aloe barbadensis* extract in the polymer matrix, which filled the foliate structure of the hydrogel as seen in the photomicrographs of the SEM. The FTIR assay corroborated, revealing the presence of bands characteristic of *Aloe barbadensis* and the hydrogel matrix forming polymers. The degree of swelling of HD-PM-EA was around 1.34-times greater than that of HD BA hydrogel. Thermal stability was observed in HD-PM-EA, and was greater in the 2% HD-PM-EA due to the higher concentration of AL. EA was effective in inhibiting the growth of two bacterial strains *S. aureus* and *Salmonella* spp. A proposed bandage is therefore produced for the treatment of chronic cutaneous lesions, in which hydrogel technology is associated with the therapeutic properties of *Aloe barbadensis* extract.

## Figures and Tables

**Table 1:** Data from the pH, density, and humidity assays of *Aloe barbadensis in natura* and extract.

Tests	*Aloe barbadensis in Natura*	*Aloe barbadensis* Extract
pH	4.19 ± 0.09	4.65 ± 0.25
Density (g/mL)	1.012 ± 0.001	1.002 ± 0.001
Humidity (%)	99.35 ± 0.162	-
Fiber Content (%)	0.6 ± 0.13	-

Results expressed as mean of triplicate ± standard deviation.

**Table 2:** Spectroscopic profile by Fourier Transform Infrared Spectroscopy (FTIR) of hydrogels.

Functional Groups		*Aloe barbadensis* Hydrogels	Basic Hydrogel
O–H stretching	Alcohols and phenols	+	+
N–H stretching	Amines	+	+
C–H stretching	Alkanes,	+	+
C=O stretching	Aldehydes and ketones	+	+
C=C stretching	Alkenes	+	–
C=C aromatics	Alkenes	+	–
C=O curves	Aldehydes and ketones	+	–
C=C curves	Alkenes	+	–
C–C curves	Alkanes	+	+
C–N stretching	Nitriles e Amines	+	+
C–O stretching	Alcohols, Aldehydes and ketones	+	+

**Table 3:** Microdilution of *Aloe barbadensis* extract for the minimum inhibitory concentration (MIC) assay.

Microdilution of *Aloe barbadensis* (µg/mL)	Gram Negative Bacteria			Gram Positive Bacteria	Gram Positive Fungi
	*Enterobacter* spp.	*E. coli*	*Salmonella entérica*	*S. aureus*	*Candida albicans*
1000	N A	NA	A	A	NA
500	NA	NA	A	A	NA
250	NA	NA	A	A	NA
125	NA	NA	A	NA	NA
62.5	NA	NA	A	NA	NA
31.25	NA	NA	NA	NA	NA

NA—No Activity; A—Activity.
